# Capping Exposed Pulps

**Published:** 1887-11

**Authors:** George H. Cushing

**Affiliations:** Chicago.


AMERICAN JOURNAL

—OF—

DENTAL SCIENCE.

VOL. XXI. THIRD SERIES—NOVEMBER, 1887. No. 7.

ARTICLE I.

### CAPPING EXPOSED PULPS.

BY GEORGE H. CUSHING, CHICAGO.

In the ten minutes allotted to this paper, the endeavor will be very briefly to outline the following points, viz.: The natural limitations of conservative treatment of the dental pulp; the reason why there is as yet so great a diversity of practice; the methods advocated by some of our best men; the necessary steps to be taken in order to secure the greatest likelihood of success, and some suggestions as to the probable cause of failure in many cases.

There would seem to be no reason, from a physiological standpoint, why exposed pulps should not be successfully treated and capped; but there is one anatomical peculiarity of these organs that places them in a somewhat different category from other organs or tissues of the body, which unquestionably limits the possibilities of conservative treatment.

This peculiarity lies in the fact that the pulp is located in a cavity with absolutely unyielding walls, while its vascular and nerve supplies enter it through a very minute opening in the same dense structure; so that any swelling, from inflammation or other causes, would produce greater or less constriction of the vessels of the pulp at the point where it is most essential that the blood and nerve supply should be uninterrupted and unimpaired.

Perhaps there is no one thing so difficult to do as to outline a comprehensive conservative treatment of exposed pulps which would be endorsed by a majority of the best men in the profession. This is a confession that there is no well-established method which is based on scientific principles, and which is justified by *general* experience.

The methods of treatment are various, as advocated by different leading men, and are followed by the mass of practitioners blindly, because of their faith in the advocate who happens to be the favorite.

The chief and perhaps only reason why there is not some well-established practice, is to be found in the fact that, as a profession, we are not scientific in our methods of observation.

We do not record our cases as we ought; we do not watch them with sufficiently critical care, and do not make sufficiently copious notes concerning them, but trust too much to memory, and *guess* too much about general results. The most conscientious observers will sometimes greatly err when trusting to memory to furnish the data upon which to base opinions.

Then again, it must be borne in mind that there are comparatively few men who are competent for scientific observation, who see all aspects of a case, and see them without bias. Some men are over-sanguine, and can see no failures—from their own hands; others are not competent to recognize a certain class of failures when they do occur; others again are dishonest, and deny failures when they know they have occurred.

Until we can secure the co-operation of a number of earnest and properly qualified men to experiment scientifically in this direction, we are likely to have no well-settled method of practice; and, were we to secure these men to do this work, years would have to elapse before their conclusions could be verified.

There are two methods which are probably generally pursued, distinguished by the names of the material used for the capping; the one, the older, being oxychloride of zinc; the other the oxyphosphate of zinc.

Oxychloride of zinc has been used more or less since introduced by Dr. Keep, of Boston, in 1858 or '59; but it came prominently to the front about 1866, through the advocacy of Dr. W. H. Atkinson, of New York, who claimed for it extraordinary success.

For some time this method was pursued very extensively, and with apparently gratifying results; but later there came to be a reaction, and so many cases that were supposed to have been successful proved otherwise in the lapse of time, that the oxychloride of zinc came to be regarded by many as the cause of these failures.

Its use was frequently attended with great pain, and many believed it to be too irritating, and that its irritating property was the inauguration of conditions which culminated in the death of the organ, though Dr. Atkinson claimed at one time that the pain and irritation were desirable features.

However, very many believed that something less irritating should be used, and it was supposed that it had been found in the oxyphosphate of zinc, and exposed pulps were capped with the latter material almost as extensively as they had before been with the oxychloride, and at first it was supposed with greater success than by the former method. Time, however, which tests all these methods inexorably, seems to have demonstrated that this material is no more to be relied upon than was the oxychloride!

These experiences very naturally suggest the inquiry whether the causes of failure are chiefly to be sought in the material used for the capping.

It is more than probable that the limitations imposed by the peculiar anatomy of the pulp before referred to, has more to do with many of these failures than the material employed.

When there has been inflammation, or even irritation of the pulp, it is veıy probable that in many cases some permanent injury is effected by reason of the constriction of the nerves and vessels passing through the minute foramina and canals, which, though seemingly cured at the time of the operation, yet develop sooner or later—sometimes after years, sometimes in a few months—a condition which results in the death of the organ. We cannot know what conditions are present in the pulp except as outward symptoms indicate; and, though a pulp may remain quiet and seemingly healthy for several years, yet its death may occur at any moment.

It may very properly be said that the capping of an exposed pulp is always an experiment—frequently justifiable, and giving strong grounds for hope in its success, but still an experiment—but the methods pursued must of course have a decided influence upon the result. The most judicious and skillfully performed operations *may* fail, but those that are careless and slipshod are quite *sure* to.

The following may briefly describe the methods to be pursued:

The tooth should be isolated by means of the rubber dam. The cavity should if possible be opened so as to give a good view of its entire interior. The debris and softened dentine should be carefully removed without impinging upon the pulp, and without producing pain, if possible—aud this is possible more frequently than many are aware.

The cavity should then be saturated with pure wood creosote, which should be allowed to remain a few moments to become absorbed, when the cavity should be very carefully dried, and a drop of Fletcher's carbolized resin should be placed over the point of exposure and left for two or three minutes, when the excess should be dried off with bibulous paper; and then the whole surface of the cavity, which is to be covered with the capping material, should be varnished with copal dissolved in ether. When this has hardened, which will be in a few minutes, the capping, either of oxychloride or oxyphosphate of zinc, should be *flowed* over the point of exposure to the depth or thickness desired. The capping material should never be *forced* into place, for injury may and is almost certain to follow any compression of the pulp. When the capping is sufficiently hardened, the filling, either temporary or otherwise, may be proceeded with. These instructions pre-suppose, of course, that the pulp is in a healthy condition.

As some of the other papers will doubtless consider quite fully the treatment of diseased pulps prior to capping, this paper will only refer to the matter in a general way.

Diseased pulps should be treated for whatever condition presents itself, on general principles, just as other tissues or organs are treated under like diseased conditions. The idea that all exposed pulp should be treated alike, or with the same remedies, is preposterous.

The causes of failures in many cases may justly be charged to the anatomical limitations before altuded to; a point which has never been sufficiently recognized, and which should always be borne in mind in any calculations as to the chances of success.

Over this cause we have no control; but there are other causes of failure, within our power to modify; and these are, want of care and thoroughness in details. It should never be forgotten that in the treatment of exposed pulps, more than in any other special operation, the least neglect or carelessness is likely to prove disastrous.

As to the chances of success in this operation, it may safely be stated that after the age of twenty-five the chances are small, even with recent exposures and healthy pulps. Under that age, all cases in which there has been inflammation, though they may seem to do well for a while, are to be regarded as liable at any time to fail; while in those cases where there has been sloughing of any portion of the pulp, failure is to be expected in a large majority of cases.—*The Dental Review.*

